# Laparoscopic-assisted Resection of a Retroperitoneal Lumbar Nerve Root Neurofibroma: A Case Report

**DOI:** 10.1055/s-0043-1770977

**Published:** 2024-01-29

**Authors:** Miguel Relvas-Silva, Estevão Rodrigues Lima, Manuel Ribeiro Silva, Nuno Neves

**Affiliations:** 1 Departamento de Ortopedia e Traumatologia, Centro Hospitalar Universitário São João, Porto, Portugal; 2 Departamento de Urologia, Hospital CUF Porto, Porto, Portugal; 3 Departamento de Ortopedia, Hospital CUF Porto, Porto, Portugal; 4 i3S - Instituto de Investigação e Inovação em Saúde, Universidade do Porto, Porto, Portugal; 5 INEB - Instituto Nacional de Engenharia Biomédica, Universidade do Porto, Porto, Portugal

**Keywords:** laparoscopy, low back pain, neurofibroma, retroperitoneal neoplasms., spinal nerve roots

## Abstract

We present a case of a 59-year-old patient with chronic low back pain, caused by a retroperitoneal intraneural tumour. Laparoscopic excision was performed and histology revealed a spinal nerve root neurofibroma. Post-operatively, the patient developed partial motor and sensitive deficits due to tumoral nerve entrapment, with progressive recovery with rehabilitation. This report reviews the literature on this sparsely reported condition, highlighting the utility of laparoscopy in its management.

## Introduction

Retroperitoneal tumours are rare and can develop from diverse structures, including retroperitoneal organs or soft tissues. 1 2 In this location, malignant tumours are roughly four times more frequent than benign lesions. 3 The clinical manifestations are nonspecific, making diagnosis and treatment challenging. 1 2 3 4

Neurofibromas are benign neurogenic tumours, representing one of the most prevalent peripheral nerve tumours. They may manifest at any age, without gender or ethnic predilection. 5 6 The majority occurs sporadically as a single nodule (less frequently, multiple lesions are identified in the setting of Neurofibromatosis type 1 - NF1) and frequently displace and encase the involved nerve roots. 5 6

Although similar in many diagnostic and treatment perspectives, they differ from schwanommas – which may compress, but rarely envelope the nerve roots. 6 7 8

## Case Report

**Fig. 1:**
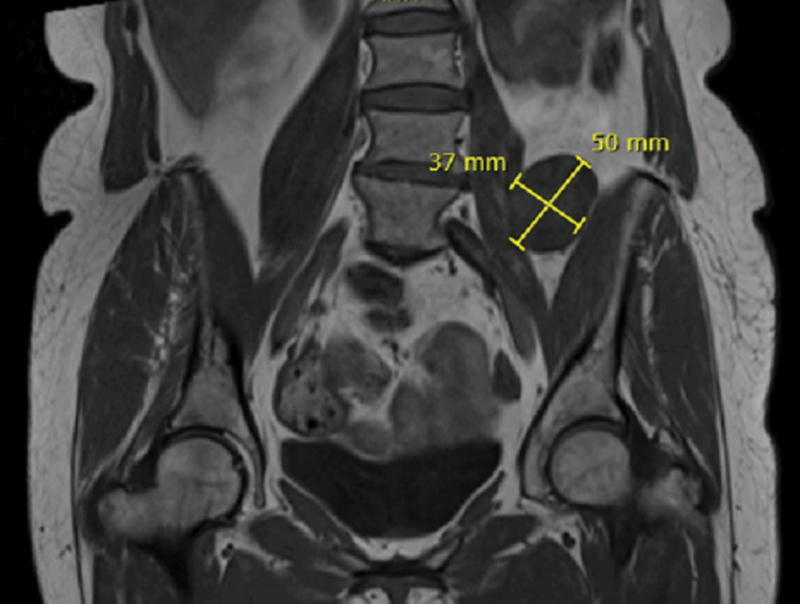
MRI (coronal plane) – T1- weighted sequence: hypointense ovoid, nodular paraspinal mass, closely related to the left L4 nerve root and located between the psoas and iliacus muscles.

59-year-old Caucasian female, with unremarkable medical/surgical history and unrelated family history, presented at the consultation with predominantly left-sided, chronic low back pain (refractory to analgesics), without leg irradiation, sensory deficits or red flags. The physical examination was unremarkable. Simple radiography showed mild degenerative spine disease. Magnetic resonance imaging (MRI) of the lumbosacral segment revealed a solid, nodular paraspinal mass (50 × 35 × 20mm), closely related to the left L4 nerve root and located between the psoas and iliacus muscles, with a well-defined cleavage plan ( Figs. 1 2 3 ) – pattern suggestive of neurogenic lumbar plexus tumour, most likely schwannoma/neurofibroma. The MRI showed no signs of disc prolapse, foraminal compression, pelvic space-occupying lesions, lymph-node enlargement or peritoneal free fluid.

**Fig. 2:**
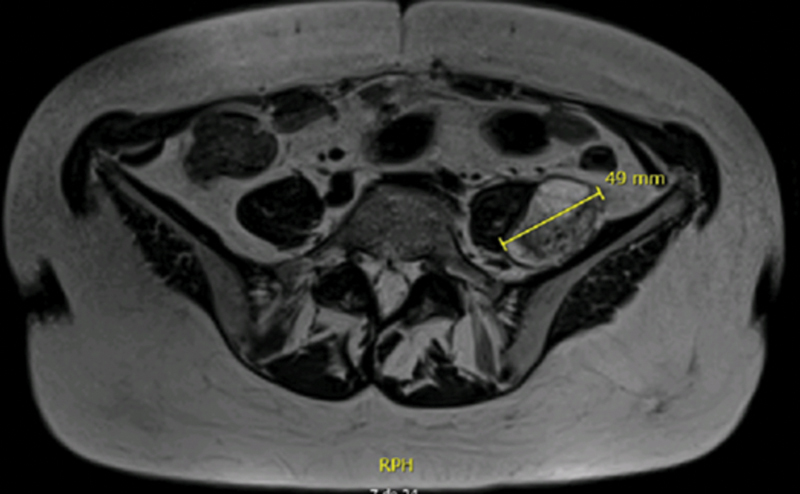
MRI (axial plane) – T2- weighted sequence: heterogeneously hyperintense lesion, with few cystic areas, suggestive of neurogenic lumbar plexus tumour.

**Fig. 3:**
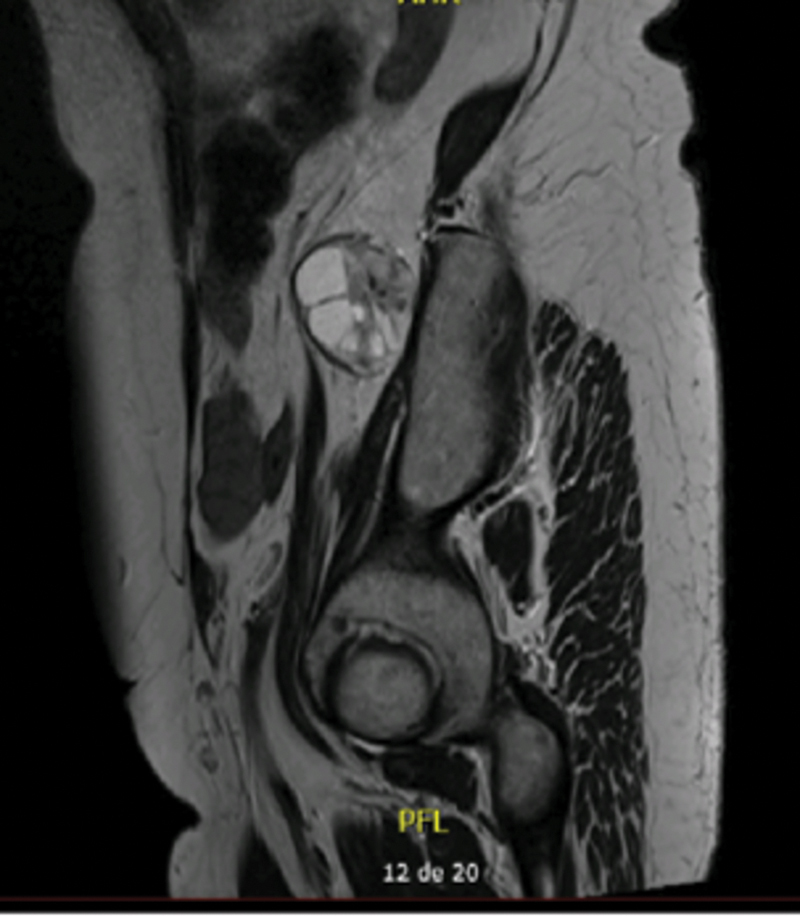
MRI (sagittal plane) – T2- weighted sequence: heterogeneously hyperintense lesion, with few cystic areas, suggestive of neurogenic lumbar plexus tumour.

Due to lesion dimension, impact on patient's quality-of-life and need of histological analysis for definitive diagnosis, surgical excision was proposed. Multidisciplinary (Orthopedics/Urology) pre-operative planning, treatment options (open *versus* laparoscopic) and complications (risk of neurological deficits) were extensively discussed with the patient. Laparoscopic excision was considered the preferred treatment modality.

### Surgical Technique *(Video Supplement)*

**Fig. 4:**
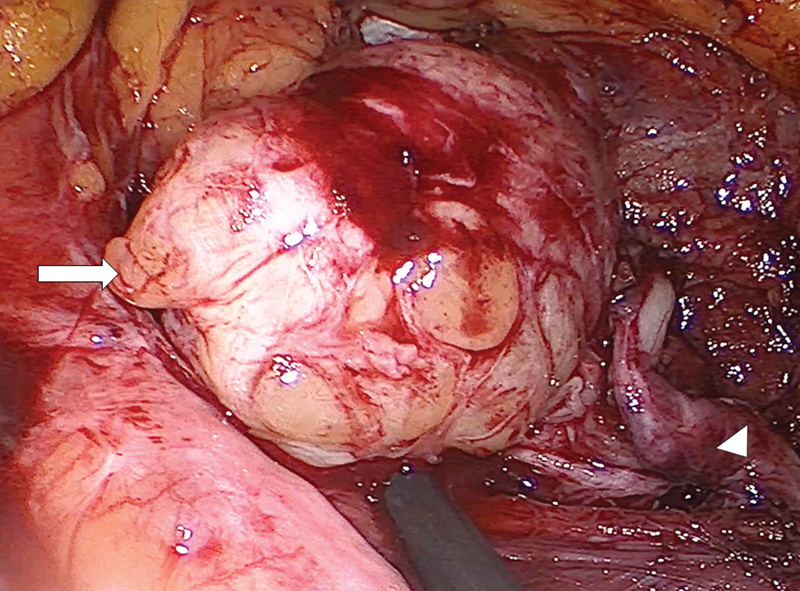
L4 nerve root neurofibroma, intermingled with the nerve fascicles. (arrow: neurofibroma; arrowhead: distal portion of the L4 nerve root).

**Fig. 5:**
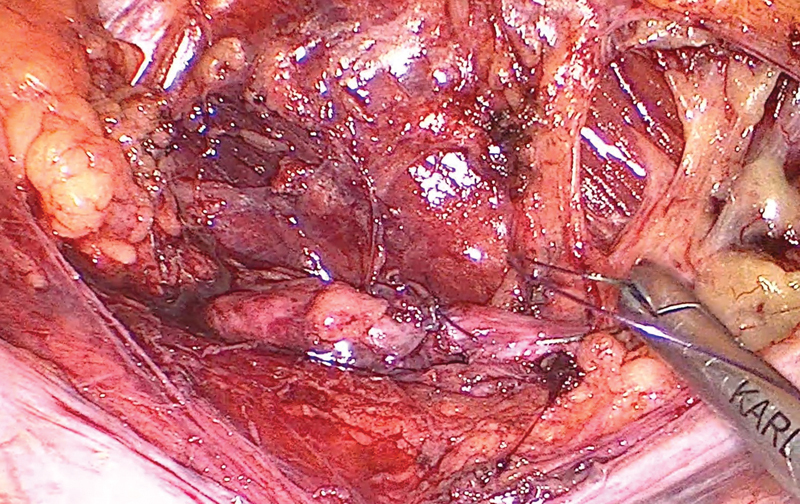
After total lesion resection with partial neurectomy, direct end-to-end epineural repair was performed.

Under general anaesthesia, the patient was placed in right semi-lateral decubitus position. Through the open laparotomy method, a 12mm-port was introduced 6cm lateral to the umbilicus. After creating a pneumoperitoneum, two 5mm ports were inserted 6cm above and 6cm below the camera, along the midclavicular line. Colon was mobilized and a retroperitoneal mass was identified. Gentle blunt dissection was performed, allowing identification of the lumbar nerve root, both proximally and distally. Epineurium was incised and numerous nerve fascicles were identified intermingled with the nodular lesion ( Fig. 4 ). Complete lesion resection was not possible without sacrificing some nerve fascicles. We decided to proceed with total lesion resection and partial nerve sacrifice. The mass was extracted *en bloc* . The epineurium was sutured ( Fig. 5 ) and a drain was inserted into the pouch-of-Douglas. Total operative time was 128 minutes (estimated blood loss: <100mL).

### Pathology

**Fig. 6:**
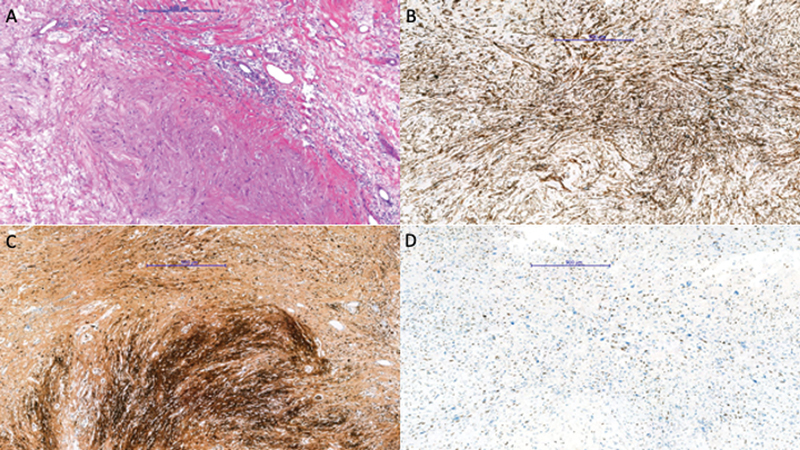
Histopathological and imunochemistry examination (5x magnification). Hematoxylin-eosin staining revealing neoplastic fusiform cells and collagenous myxoid stroma (A), with positive CD34 (B), S100 (C) and SOX10 (D) immunoreactivity.

The surgical specimen consisted of a grey roundish mass, heterogeneously yellow on cut, weighting 18.7 grams. Histopathological examination ( Fig. 6A ) showed a neoplasm of fusiform cells, with low to moderate cellularity and abundant collagenous myxoid stroma. Neoplastic cells had elongated nucleus, moderate pleomorphism, very low mitotic activity, no significant atypia or necrotic areas. Imunochemistry identified S100+ and SOX10+ cells, with CD34+ lesional cells ( Fig. 6B-D ). Epithelial membrane antigen (EMA)+ cells were only present in peripheral perineural cells and no expression of desmin or actin was detected. These findings suggest a neurofibroma. Moreover, neither Antoni A cell population, nor Verocay bodies were detected, differentiating it from schwannomas

### Follow-up

The patient woke-up with partial motor (grade 4 active knee extension and ankle dorsiflexion) and sensory (leg hypoesthesia) left lower-limb deficits, consistent with L4 nerve root lesion. The immediate postoperative phase underwent without any other major complications and excellent pain control. The patient was discharged home on day 3, with walking crunches and, one week later initiated an outpatient rehabilitation protocol. At six months, she was fully ambulatory, with complete motor recovery, although complaining of minimal knee instability (probably related to mild quadriceps atrophy) and residual leg hyposthesia. At one-year follow-up, she was feeling extremely satisfied with the outcome.

## Discussion

There is very limited data on the epidemiology, presentation, diagnosis, treatment and recurrence rate of retroperitoneal sporadic intraneural neurofibromas.

Neurofibromas have three main morphologic forms: 1) *cutaneous* , the most frequent; 2) *intraneural* , circumscribed to a peripheral nerve; or 3) *plexiform* , involving multiple fascicles of a major trunk/plexus, pathognomonic of NF1. 5 6 In terms of location, multiple bilateral spinal nerve roots tumours are hallmark of NF1, while involvement of spinal roots in sporadic forms is rare. 6

Due to slow expansion, most patients are asymptomatic and diagnosed incidentally. 5 When symptomatic, they may present with unspecific symptoms or sometimes motor/sensory deficits in relation with the affected nerve root. 6

Differential diagnosis of primary retroperitoneal masses in adults may include both neoplastic and non-neoplastic lesions. 4 8 Imaging is helpful in diagnosis. Computer tomography (CT) and magnetic resonance imaging allow the characterization of retroperitoneal lesions. On CT scan, neurofibroma presents as a well-defined mass, hypodense relative to muscle, with little or no contrast enhancement. On MRI, it has low to intermediate signal intensity on T1-weighted sequence and high signal sequence on T2-weighted images (sometimes with a characteristic but not pathognomonic *target sign* ). 9 Definitive diagnosis is ultimately based on histological examination. According to the World Health Organization Classification of Tumours of the Central Nervous System, neurofibromas correspond to grade I tumours. 6 They feature a mixture of Schwann cells, nerve axons and fibroblastic, perineurial-like and inflammatory cells, lacking Antoni A cells and Verocay bodies ( *versus* schwannomas). Immunohistochemistry shows strong expression of S100 protein (in a lower proportion than schwannomas). CD34 is variably positive in fibroblastic-like cells and EMA is focally positive in entrapped perineurial-like cells (despite lacking the diffuse staining pattern seen in perineurinomas). 5

Complete local tumour excision should be regarded as the treatment of choice. Due to location, complexity and possible nerve entrapment, treatment requires thorough preoperative planning and multidisciplinary approach. 10 11 Tumoral nerve entrapment may make it impossible to remove the lesion without sacrificing the nerve – this is responsible for the high prevalence of root resection in neurofibromas (versus schwannomas, which initially grow eccentrically, dislocating the fascicles that are not part of the diseased tissue). 3 12

Surgical removal of retroperitoneal soft-tissue tumours may include both open and laparoscopic approaches. The latter is safe and efficient, allowing direct high-definition vision, better operative field exposure in a narrow working space, accurate dissection and reduced risk of inadvertent vascular/neural injury. 11 13 Besides, its minimally invasiveness allows for faster recovery, pain control, early hospital discharge and excellent cosmetic results. 13

Although the recurrence rate and malignant transformation of neurofibromas are low (except for the plexiform subtype), follow-up is required for management of potential complications and surveillance. 5 6 Regarding this topic, to the best of our knowledge, there are no clear recommendations, except for patients with NF1. 14 Therefore, after a complete resection, we consider adequate to follow the patient clinically, considering imaging (MRI, CT or Positron emission tomography scan) and/or electromyography, when there is clinical suspicion of incomplete resection, relapse or recurrence.

This report is unique as it presents a case of chronic low back pain, due to a retroperitoneal nerve root neurofibroma – a rare benign tumour, few times identified in this location. Diagnosis is challenging and therapeutic considerations may be discussed with the patient, considering the risk of iatrogenic neurological deficits.
