# Supplementary material for: Laparoscopic-assisted Resection of a Retroperitoneal Lumbar Nerve Root Neurofibroma: A Case Report
Source: Rev Bras Ortop (Sao Paulo). 2024 Jan 29;59(Suppl 1):e109–13. doi: 10.1055/s-0043-1770977 (PMC11254426; doi:10.1055/s-0043-1770977)
Supplement: Related texts

# Research article: Ressecção assistida por laparoscopia de neurofibroma retroperitoneal da raiz nervosa lombar: Relato de caso

**Keywords:** dor lombar, laparoscopia, neoplasias retroperitoneais, neurofibroma, raízes nervosas espinhais

## Abstract

Apresentamos o caso de uma paciente de 59 anos com lombalgia crônica causada por tumor intraneural retroperitoneal. A excisão laparoscópica foi realizada e a histologia revelou um neurofibroma da raiz do nervo espinhal. No período pós-operatório, a paciente desenvolveu déficits motores e sensitivos parciais devido ao encarceramento do nervo tumoral, com recuperação progressiva à reabilitação. Este relato revisa a literatura sobre essa doença pouco descrita, destacando a utilidade da laparoscopia em seu tratamento.

## Introdução

Os tumores retroperitoneais são raros e podem ser originários de diversas estruturas, inclusive órgãos retroperitoneais ou tecidos moles. 1 2 Nessa localização, os tumores malignos são cerca de quatro vezes mais frequentes do que as lesões benignas. 3 As manifestações clínicas são inespecíficas, tornando o diagnóstico e o tratamento desafiadores. 1 2 3 4

Os neurofibromas são tumores neurogênicos benignos e representam um dos tumores de nervos periféricos de maior prevalência. Podem se manifestar em qualquer idade, sem predileção por sexo ou etnia. 5 6 A maioria ocorre esporadicamente como um nódulo único (com menor frequência, lesões múltiplas são identificadas como neurofibromatose de tipo 1 [NF1]) e tendem a se deslocar e envolver as raízes do nervo acometido. 5 6

Embora semelhantes em muitos aspectos diagnósticos e terapêuticos, estes tumores diferem dos schwannomas, que podem comprimir, mas raramente envelopam as raízes nervosas. 6 7 8

## Relato de Caso

Mulher caucasiana de 59 anos, com histórico médico/cirúrgico normal e histórico familiar não relacionado, veio à consulta com dor lombar crônica predominantemente do lado esquerdo (refratária a analgésicos), sem irradiação para as pernas, déficits sensoriais ou sinais alarmantes. Os achados do exame físico eram normais. A radiografia simples mostrou doença degenerativa branda da coluna. A ressonância magnética (RM) do segmento lombossacral revelou uma massa paraespinhal sólida e nodular (50 × 35 × 20 mm), intimamente relacionada à raiz nervosa de L4 do lado esquerdo e localizada entre os músculos psoas e ilíaco, com plano de clivagem bem definido ( Figs. 1 2 3 ). Este era um padrão sugestivo de tumor neurogênico do plexo lombar, provavelmente schwannoma/neurofibroma. A RM não mostrou sinais de prolapso de disco, compressão de foramina, lesões que ocupassem o espaço pélvico, linfoadenomegalia ou fluido livre na cavidade peritoneal.

**Fig. 1:** Ressonância magnética (plano coronal). A sequência ponderada em T1 releva uma massa nodular paraespinhal ovoide hipointensa, bastante próxima à raiz nervosa de L4 e localizada entre os músculos psoas e ilíaco.

**Fig. 2:** Ressonância magnética (plano axial). A sequência ponderada em T2 mostra uma lesão de hiperintensidade heterogênea, com poucas áreas císticas, sugestiva de tumor neurogênico do plexo lombar.

**Fig. 3:** Ressonância magnética (plano sagital). A sequência ponderada em T2 mostra uma lesão de hiperintensidade heterogênea, com poucas áreas císticas, sugestiva de tumor neurogênico do plexo lombar.

Devido à dimensão da lesão, ao impacto na qualidade de vida da paciente e à necessidade de análise histológica para diagnóstico definitivo, a excisão cirúrgica foi proposta. O planejamento pré-operatório multidisciplinar (Ortopedia/Urologia), as opções terapêuticas (procedimento aberto ou laparoscópico) e as complicações (risco de déficits neurológicos) foram amplamente discutidas com a paciente. A excisão laparoscópica foi considerada a modalidade de tratamento preferida.

## Técnica Cirúrgica *(Vídeo Suplementar)*

Sob anestesia geral, a paciente foi colocada em decúbito semilateral direito. Por meio de laparotomia aberta, um portal de 12 mm foi introduzido 6 cm lateralmente ao umbigo. Depois de criar um pneumoperitônio, dois portais de 5 mm foram inseridos 6 cm acima e 6 cm abaixo da câmera, ao longo da linha hemiclavicular. O cólon foi mobilizado e uma massa retroperitoneal foi identificada. A dissecção romba delicada foi realizada, permitindo a identificação da raiz nervosa lombar, tanto proximal quanto distalmente. O epineuro foi incisado e numerosos fascículos nervosos foram identificados entremeados à lesão nodular ( Fig. 4 ). A ressecção completa da lesão não foi possível sem o sacrifício de alguns fascículos nervosos. Optou-se pela ressecção total da lesão e sacrifício parcial do nervo. A massa foi extraída em bloco. O epineuro foi suturado ( Fig. 5 ) e um dreno foi inserido na bolsa de Douglas. O tempo cirúrgico total foi de 128 minutos (perda sanguínea estimada: <100mL).

**Fig. 4:** Neurofibroma da raiz nervosa de L4 entremeada aos fascículos nervosos (seta: neurofibroma; ponta de seta: porção distal da raiz nervosa de L4).

**Fig. 5:** Após a ressecção total da lesão com neurectomia parcial, o reparo das extremidades do epineuro foi realizada.

## Patologia

A peça cirúrgica consistia em uma massa arredondada cinza, heterogeneamente amarelada ao corte, com peso de 18,7 gramas. O exame histopatológico ( Fig. 6A ) mostrou uma neoplasia de células fusiformes, com celularidade baixa a moderada e abundante estroma mixoide colagenoso. As células neoplásicas apresentavam núcleo alongado, pleomorfismo moderado e atividade mitótica muito baixa, sem atipias significativas ou áreas de necrose. A imunoquímica identificou células S100+ e SOX10 + , com células lesionais CD34+ ( Fig. 6B-D ). A positividade para o antígeno de membrana epitelial (EMA) foi observada apenas em células perineurais periféricas e não houve deteção de expressão de desmina ou actina. Esses achados sugerem um neurofibroma. Além disso, populações de células Antoni A e corpos de Verocay não foram observados, diferenciando a lesão de um schwannoma.

**Fig. 6:** Exame histopatológico e imunoquímico (aumento: 5x). A coloração com hematoxilina-eosina revelou células neoplásicas fusiformes e estroma mixoide colagenoso (A), com imunorreatividade positiva para CD34 (B), S100 (C) e SOX10 (D).

## Acompanhamento

A paciente despertou com déficit motor parcial (grau 4 de extensão ativa do joelho e dorsiflexão do tornozelo) e sensorial (hipoestesia da perna) no membro inferior esquerdo, condizente com lesão da raiz nervosa de L4. O período pós-operatório imediato transcorreu sem maiores intercorrências e com excelente controle da dor. A paciente teve alta no terceiro dia, andando com muletas, e, uma semana depois, iniciou um protocolo de reabilitação ambulatorial. Aos seis meses, caminhava sem auxílio e apresentava recuperação motora completa, embora se queixasse de instabilidade mínima do joelho (provavelmente relacionada à atrofia branda do quadríceps) e hipoestesia residual da perna. Após um ano de acompanhamento, a paciente estava extremamente satisfeita com o resultado.

## Discussão

Os dados sobre a epidemiologia, quadro clínico, diagnóstico, tratamento e taxa de recorrência de neurofibromas intraneurais esporádicos retroperitoneais são muito limitados.

Há três formas morfológicas principais de neurofibromas: 1) *cutânea* , a mais frequente; 2) *intraneural* , circunscrita a um nervo periférico; ou 3) *plexiforme* , com acometimento de múltiplos fascículos de um tronco/plexo maior, patognomônico de NF1. 5 6 Quanto à localização, múltiplos tumores bilaterais de raízes nervosas espinhais são característicos de NF1, enquanto o acometimento de raízes espinhais em formas esporádicas é raro. 6

Devido à expansão lenta, a maioria dos pacientes é assintomática e diagnosticada de maneira incidental. 5 Quando sintomáticos, os pacientes podem apresentar sintomas inespecíficos ou, às vezes, déficits motores/sensoriais relacionados à raiz nervosa envolvida. 6

O diagnóstico diferencial de massas retroperitoneais em adultos pode incluir lesões neoplásicas e não neoplásicas. 4 8 As técnicas de diagnóstico por imagem auxiliam o diagnóstico. A tomografia computadorizada (TC) e a RM permitem a caracterização das lesões retroperitoneais. À TC, o neurofibroma é uma massa bem definida, hipodensa em relação ao músculo, com pouco ou nenhum realce por contraste. À RM, apresenta intensidade de sinal baixa a intermediária na sequência ponderada em T1 e sequência de alto sinal nas imagens ponderadas em T2 (às vezes com um sinal em alvo [ *target sign* ] característico, mas não patognomônico). 9 O diagnóstico definitivo é, em última análise, baseado no exame histológico. De acordo com a Classificação de Tumores do Sistema Nervoso Central da Organização Mundial da Saúde, os neurofibromas são tumores de grau I. 6 Estes tumores apresentam uma mistura de células de Schwann, axônios nervosos e células fibroblásticas, perineuriais e inflamatórias, sem células Antoni A e corpos de Verocay (diferentemente dos schwannomas). A imuno-histoquímica mostra forte expressão da proteína S100 (em menor proporção que os schwannomas). CD34 é variavelmente positivo em células semelhantes a fibroblastos e EMA é focalmente positivo em células semelhantes a perineuros aprisionados (apesar da ausência do padrão de coloração difusa observado nos perineuriomas). 5

A excisão local completa do tumor deve ser considerada o tratamento de escolha. Devido à localização, complexidade e possível encarceramento do nervo, o tratamento requer planejamento pré-operatório minucioso e abordagem multidisciplinar. 10 11 O encarceramento do nervo tumoral pode impossibilitar a retirada da lesão sem sacrifício do nervo, o que é responsável pela alta prevalência de ressecção radicular em neurofibromas (diferentemente dos schwannomas, que, a princípio, crescem de maneira excêntrica, deslocando os fascículos que não fazem parte do tecido doente). 3 12

A remoção cirúrgica de tumores de tecidos moles retroperitoneais pode ser feita por abordagens abertas ou laparoscópicas. Estas últimas são seguras e eficientes, permitindo a visualização direta de alta definição, a melhor exposição do campo operatório em um pequeno espaço de trabalho, a dissecção precisa e o menor risco de lesão vascular/neurológica inadvertida. 11 13 Além disso, sua invasividade mínima permite a recuperação mais rápida, o controle da dor, a alta hospitalar precoce e excelentes resultados cosméticos. 13

Embora a taxa de recorrência e transformação maligna de neurofibromas seja baixa (à exceção do subtipo plexiforme), há necessidade de acompanhamento para o manejo de possíveis complicações e vigilância. 5 6 Em relação a isso, até onde temos conhecimento, não há recomendações claras, exceto para pacientes com NF1. 14 Portanto, após a ressecção completa, consideramos adequado o acompanhamento clínico do paciente, incluindo exames de imagem (RM, TC ou tomografia por emissão de pósitrons) e/ou eletromiografia em caso de suspeita clínica de ressecção incompleta, recorrência ou recidiva.

Este relato é inédito por apresentar um caso de lombalgia crônica, decorrente de neurofibroma retroperitoneal de raiz nervosa – um tumor benigno raro, poucas vezes identificado nesta localização. O diagnóstico é desafiador e as considerações terapêuticas podem ser discutidas com o paciente, inclusive o risco de déficits neurológicos iatrogênicos.
